# Signal automatic modulation based on AMC neural network fusion

**DOI:** 10.1371/journal.pone.0304531

**Published:** 2024-06-06

**Authors:** Haoran Yin, Junqin Diao

**Affiliations:** 1 School of Computer Science and Engineering, Sun Yat-Sen University, Guangzhou, China; 2 School of Computer Science, Durham University, Durham, England, United Kingdom; Shandong Normal University, CHINA

## Abstract

With the rapid development of modern communication technology, it has become a core problem in the field of communication to find new ways to effectively modulate signals and to classify and recognize the results of automatic modulation. To further improve the communication quality and system processing efficiency, this study combines two different neural network algorithms to optimize the traditional signal automatic modulation classification method. In this paper, the basic technology involved in the communication process, including automatic signal modulation technology and signal classification technology, is discussed. Then, combining parallel convolution and simple cyclic unit network, three different connection paths of automatic signal modulation classification model are constructed. The performance test results show that the classification model can achieve a stable training and verification state when the two networks are connected. After 20 and 29 iterations, the loss values are 0.13 and 0.18, respectively. In addition, when the signal-to-noise ratio (SNR) is 25dB, the classification accuracy of parallel convolutional neural network and simple cyclic unit network model is as high as 0.99. Finally, the classification models of parallel convolutional neural networks and simple cyclic unit networks have stable correct classification probabilities when Doppler shift conditions are introduced as interference in practical application environment. In summary, the neural network fusion classification model designed can significantly improve the shortcomings of traditional automatic modulation classification methods, and further improve the classification accuracy of modulated signals.

## 1. Introduction

With the rapid modern communication technologies growth, wireless signal modulation modes are becoming increasingly diverse, making signal detection and classification particularly important. Signal modulation classification is a fundamental problem in communication fields, involving correctly identifying transmitted signals for ensuring data transferring accuracy and reliability [1]. To address this challenge, many scholars have done researches to obtain efficient and accurate methods for signal modulation classification [2]. Automatic Modulation Classification (AMC) technology, as a key solution for signal automatic modulation, acts as a crucial factor in ensuring the security of wireless communication [3, 4]. Traditional signal modulation recognition methods, such as statistical methods based on feature extraction, can achieve good classification results under certain conditions, but their performance is often limited when facing complex communication environments and diverse modulation modes. As a milestone of deep learning technology in multiple industries, neural networks have become powerful tools to solve such problems. Recently, it reached prominent outcomes in areas like image recognition and speech process. Convolutional Neural Networks (CNNs) are widely used in related image and video analysis works due to their powerful feature extraction ability. Additionally, Recurrent Neural Networks (RNNs) also exhibit unique advantages in sequence data processing. Currently, most researches on signal modulation classification focus on traditional learning ways [5]. Although these methods may work well in some scenarios, they struggle to handle complex and high-dimensional signal data. In contrast, combining different neural network algorithms is theoretically possible. It can be expected that it overcomes the mentioned issues and becomes a more powerful and flexible solution for signal modulation classification. Based on this background, this study innovatively combines CNNs with RNNs and designs three different connection ways by changing the positions of the two neural networks, namely Convolutional Neural Network-Simple Recurrent Unit (CNN-SRU), Simple Recurrent Unit-Convolutional Neural Network (SRU-CNN), and CNN Parallel SRU (CPS). Accordingly, three types of signal AMC models are built. By combining the advantages of multiple neural network models, these models can more effectively process multiple types of signal data and improve the accuracy and robustness of signal modulation classification. Compared with single neural network models, the fused algorithms can provide more comprehensive and deeper feature learning and data representation. Finally, a series of experimental tests is conducted to verify the superiority of the designed models and identify the best connection approach. This study contains 5 parts. The first displays a brief statement on the full-text content. The second part analyzes and summarizes relevant research conducted by others. The third part focuses on how to build signal AMC models. The fourth part evaluates the performance and application effects of various models. The fifth part provides a summary of the full text.

## 2. Related work

CNNs and RNNs are both commonly used neural network structures in deep learning. Each network has its own characteristics and applications. For the problem of fingerprint and background segmentation in potential fingerprint images, M. Chhabra et al. proposed an early distinction technology based on color and significance masks, and combined CNNs to perform preliminary distinction of the images. CNNs were used to distinguish between false fingerprint areas and real fingerprint areas through optimization, while stacked auto-encoders provided better features for the CNNs. The research outcomes displayed that the designed image segmentation model achieved a segmentation accuracy of 98.45% on high-quality fingerprint images [6]. To address the issue of low recognition accuracy of existing radar signal recognition techniques, W. Si et al. proposed an efficient method combining deep CNNs and fusion methods. Two deep CNN models were constructed for extracting features with high efficiency. The experiment outcomes displayed an 84.38% mean recognizing accuracy at a Signal-to-Noise Ratio (SNR) of -12 dB. It even reached 94.31% at an SNR of -10 dB. Especially in low SNR environments, the recognition accuracy of this method was significantly better than others [7]. For bringing improvement to the manual detection of exudates in fundus images, E. Dhiravidachelvi et al. proposed a new image detection method combining CNN-RNN with an artificial bee colony optimization algorithm. This method first used Hough transformation to remove the optic disc to avoid false positives. Then, color and texture features were extracted from fundus images to distinguish between exudates and non-exudates. Finally, this method was used for classification. Experiment outcomes displayed that the proposed image classification method performed a accuracy of up to 97.4% [8]. A. Das et al. proposed a method for enhancing the accuracy of handwritten digit recognition. Firstly, 1000 images were generated with a generative adversarial CNN to increase the size of the dataset. Then, differed network units were combined for image classification. Finally, to obtain the minimized error, Adam optimization was used, and a supervised learning approach was activated for the network training. The research outcomes displayed a recognition accuracy of 98.32% [9].

AMC is categorized as a wireless communicating tech that automatically identifies and classifies the received signal modulation type. In many communication applications, knowledge of the received signal modulation mode is crucial. Currently, many scholars have conducted a series of optimization studies on traditional AMC techniques. To address the problem of dispersed training data for signal modulation classification methods on the network, X. Fu et al. proposed a method that combined decentralized learning and ensemble learning. It was named as DeEnAMC. The research results showed that this method not only reduced communication overhead but also had better signal classification performance [10]. To reduce the training samples provided by AMC methods for each modulation type, thereby improving the performance of AMC methods in practical applications, J. Che et al. proposed a novel spatiotemporal mixed feature extraction network to optimize traditional AMC methods. The research results showed that this feature extraction framework could promote the classification of spatial signals, thereby improving the effectiveness and robustness of signal modulation classification [11]. S. Ying et al. proposed a data-driven framework based on CNNS and transformers. This framework was applied to AMC techniques to achieve the improvement of the received signal modulation effect. Extensive simulation experiments demonstrated that the data-driven framework had its capability to efficiently enhance classification accuracy and classification time, thereby enhancing AMC effectiveness [12]. M. H. Essai et al. developed an AMC scheme based on CNNs with the aim of optimizing the modulation capability and generalization ability of current limited signal datasets. The proposed AMC scheme could achieve signal classification without feature extraction and had self-learning feature capabilities. The research outcomes displayed that the proposed AMC technology could achieve faster signal classification under different SNR conditions [13].

As a pivotal technology in wireless communication systems, AMC is employed to automatically identify the modulation type of the received signal. Deep learning facilitates feature extraction and learning by constructing intricate model structures and leveraging vast quantities of data for training. Currently, numerous experts have leveraged deep learning models to address signal modulation challenges [14]. D. Xu et al. presented a framework for Undetectable Universal Counterperturbations (UAPs) in AMC systems. The framework employed a custom loss function and wavelet reconstruction to simplify a variety of adversarial attacks, significantly reducing the accuracy and performance of the model on two radio signal datasets. The resulting class-differentiation attacks resulted in a 58.20% reduction in model accuracy on average, while target-specific attacks resulted in a 73.78% reduction in performance [15]. R. Zhang et al. introduced a novel class-discovery method for AMC. This method addressed the challenge of class-disjoint environments, where AMC models need to identify new types of modulation. The method utilized a three-stage deep learning approach to distinguish between known classes and cluster new class samples. The approach utilized common knowledge extracted from feature similarities in a labeled training dataset. Simulation results confirmed the effectiveness and improved performance of the proposed method in identifying and classifying new modulation classes [16]. In a two-stage data enhancement method using spectral interference for AMC, Q. Zheng et al. exploited the temporal frequency variability inherent in disparate radio signals. Their approach involved reconstructing the enhanced signal through inverse Fourier transform and subsequently employing it alongside the original signal for training and testing purposes. By incorporating the enhanced signal into the network, the generalization capacity of the network was enhanced. Experiments on the RadioML 2016.10a dataset demonstrated a significant improvement over traditional methods and state-of-the-art AMC techniques [17].

In summary, most of the current research on CNNS and RNNS is focused on the field of images, including image recognition and object detection. The study of AMC is mostly limited to optimizing with some specific neural networks, there are few studies on optimizing by using fusion networks. In this context, this study combines two different neural network algorithms to build models, and ultimately builds three types of signal AMC models based on different fusion methods, aiming to further enrich the research and application of neural networks in signal classification.

## 3 Design of signal AMC model with neural network fusion

In real wireless communication scenarios, there are various factors that may affect signals, like multi-path, blocking, noise, etc. Therefore traditional AMC technology needs update to enhance its performance. With the advancement of machine learning and deep learning technologies, this study combines CNNs with RNNs and designs three types of neural network fusion models using three different connection methods to achieve signal AMC through this model.

### 3.1 Signal automatic modulation

Modulation is categorized as a technology for processing signals, that mainly converts an information signal into a form that is suitable for transmission [18]. In communication, modulation usually involves changing some attributes of a carrier signal, such as the amplitude, frequency, or phase of the carrier signal. By changing its attributes, the carrier signal can carry and transmit information [19]. Modulation is not only common in wireless communication but also widely used in wired communication, such as telephone lines, optical fibers and other transmission media. The main purpose of modulation is to ensure that information signals can be reliably and efficiently transmitted in various transmission environments. The current commonly used signal modulation methods are digital and analog modulation.

Amplitude Modulation (AM) belongs to the analog category. The purpose of this technology is to complete the transmission of the modulated signal information under the condition of a constant frequency. This purpose is achieved by varying the carrier signal amplitude with the changes of the modulation signal. The amplitude-modulated signal expression is shown in Eq (1) [20].


$$s_{AM}(t)=A_{c}[1+m(t)]\cos(2\pi f_{c}t+\theta_{0})$$ (1)

In Eq (1), *s*_*AM*_(*t*) means the amplitude-modulated signal at time t. *A*_*c*_ means the amplitude of the carrier signal. *f*_*c*_ means the frequency of the carrier signal. *θ*_0_ means the initial phase of the signal. *m*(*t*) means the modulation signal. *t* means time. In addition to AM, angle modulation is also a type of analog modulation. Common types of angle modulation include frequency-modulated signals and phase-modulated signals.

Compared with analog modulation, digital modulation has better anti-interference properties and is more secure. In modern communication fields, digital modulation can handle complex and diverse signal problems. Common digital modulation types can be divided into Phase Shift Keying (PSK), Frequency Shift Keying (FSK), Quadrature Amplitude Modulation (QAM), and Pulse Amplitude Modulation (PAM). In multi-order digital modulation systems, the value of the modulation signal M takes different values, which is shown in Eq (2) [21].


$$m(t)=M=2^{k}$$ (2)

In Eq (2), *M* represents the value taken by the modulation signal in a multi-order digital modulation system. *k* represents a positive integer. In multi-order digital modulation systems, the specific mathematical expression of the modulation signal is shown in Eq (3)

$$s(t)=\gamma \cos(2\pi f_{c}t+\theta_{0})+\lambda \sin(2\pi f_{c}t+\theta_{0})$$ (3)

In Eq (3), *s*(*t*) represents the multi-order digital modulation signal at time t. *γ* and *λ* both represent modulation parameters.

### 3.2 Signal modulation classification model

With the continuous development of CNNs and RNNs, they have become a commonly used solution to solve various wireless communication problems, including signal AMC. AMC is a technology that automatically identifies and classifies signal modulation types in communication systems, which can improve receiver performance and enhance spectrum perception capability in wireless communication. Traditional AMC relied on manually designed features, resulting in unsatisfactory performance. By combining CNN and RNN, AMC can automatically learn and extract features from data, thereby avoiding complex feature extraction engineering and making signal feature classification more efficient and accurate.

**Fig 1:**
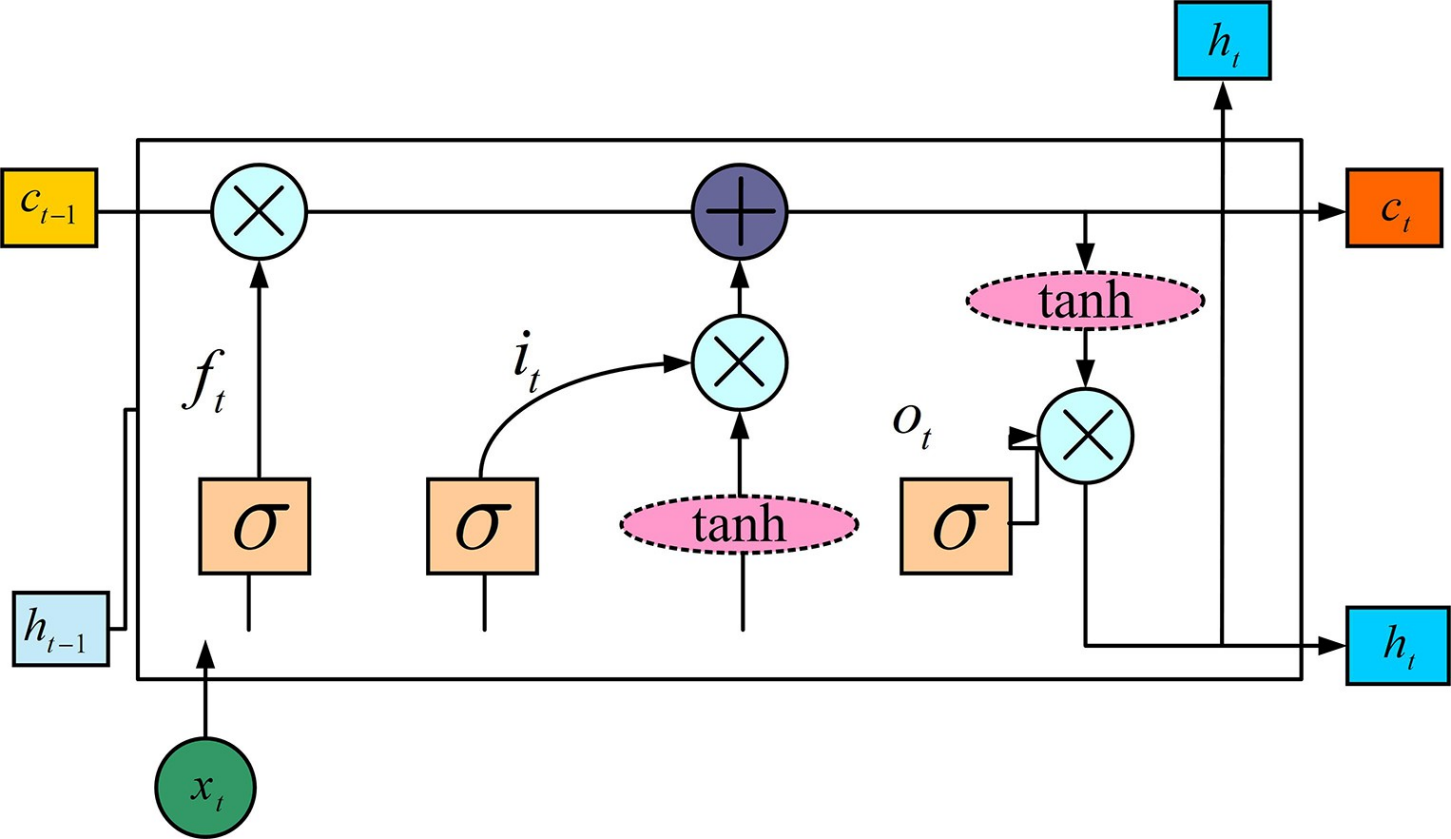
Structure of LSTM neuron.

Long Short-Term Memory (LSTM) is a special architecture of RNN proposed by Sepp Hochreiter and Jürgen Schmidhuber in 1997 [22]. LSTM can be used to effectively solve the problems of gradient disappear and gradient explosion that traditional RNN encounters when learning long sequences. Due to LSTM’s good sequence processing ability, this network structure is also widely used in the optimization of AMC. The neuron structure of LSTM is shown in Fig 1.

The LSTM shown in Fig 1 mainly contains gates of output, input, and forget. The input gates decide which information is going to be updated in the memory unit. The forget gates decide whether the information in the memory unit needs to be forgotten or discarded. Finally, the output gate is used to output the screened information, which will be used as the input of the next unit. The calculation formula of the forget gate is shown in Eq (4).


$$f_{t}=\sigma (W_{f}\cdot [h_{t-1},x_{t})]+b_{f}$$ (4)

In Eq (4), *f*_*t*_ is the forget gate, *b*_*f*_ means the bias vector belongs to it, *h*_*t*−1_ means the value of the hidden layer at time *t*−1, *W*_*f*_ means the weight matrix of the forget gate, and *x*_*t*_ means the input of the previous unit at time *t*. *σ* means the activation function, which is utilized with the function of Sigmoid. The calculation formula of the input gate is shown in Eq (5).


$$i_{t}=\sigma (W_{i}\cdot [h_{t-1},x_{t}]+b_{i})$$ (5)

In Eq (5), *i*_*t*_ means the input gate, *W*_*i*_ is the input gate matrix of weight, and *b*_*i*_ means the bias vector of the input gate. Eq (6) gives the output calculating mathematical expression.


$$o_{t}=\sigma (W_{o}\cdot [h_{t-1},x_{t}]+b_{o})$$ (6)

In Eq (6), *o*_*t*_ means the output gate, *W*_*o*_ means the weight matrix of the output gate, and *b*_*o*_ means the bias vector of the output gate. The calculation formula of the cell state is shown in Eq (7).


$$c_{t}=f_{t}⊙c_{t-1}+i_{t}⊙s_{t}$$ (7)

In Eq (7), *c*_*t*_ means the cell state of this unit at time *t*. *c*_*t*−1_ means the cell state at the previous moment, and ⊙ means the Hadamard product operation. *s*_*t*_ is calculated after passing through the tanh function, and it acts as an output from the memory unit. The calculation process is shown in Eq (8).


$$s_{t}=\tanh(W_{c}\cdot [h_{t-1},x_{t}]+b_{c})$$ (8)

In Eq (8), *W*_*c*_ means the weight matrix of the input gate after passing through the tanh function, and *b*_*c*_ means the input bias vector under this circumstance. Finally, the output value of this neuron is *h*_*t*_, which is shown in Eq (9).


$$h_{t}=o_{t}⊙\tanh(c_{t})$$ (9)

**Fig 2:**
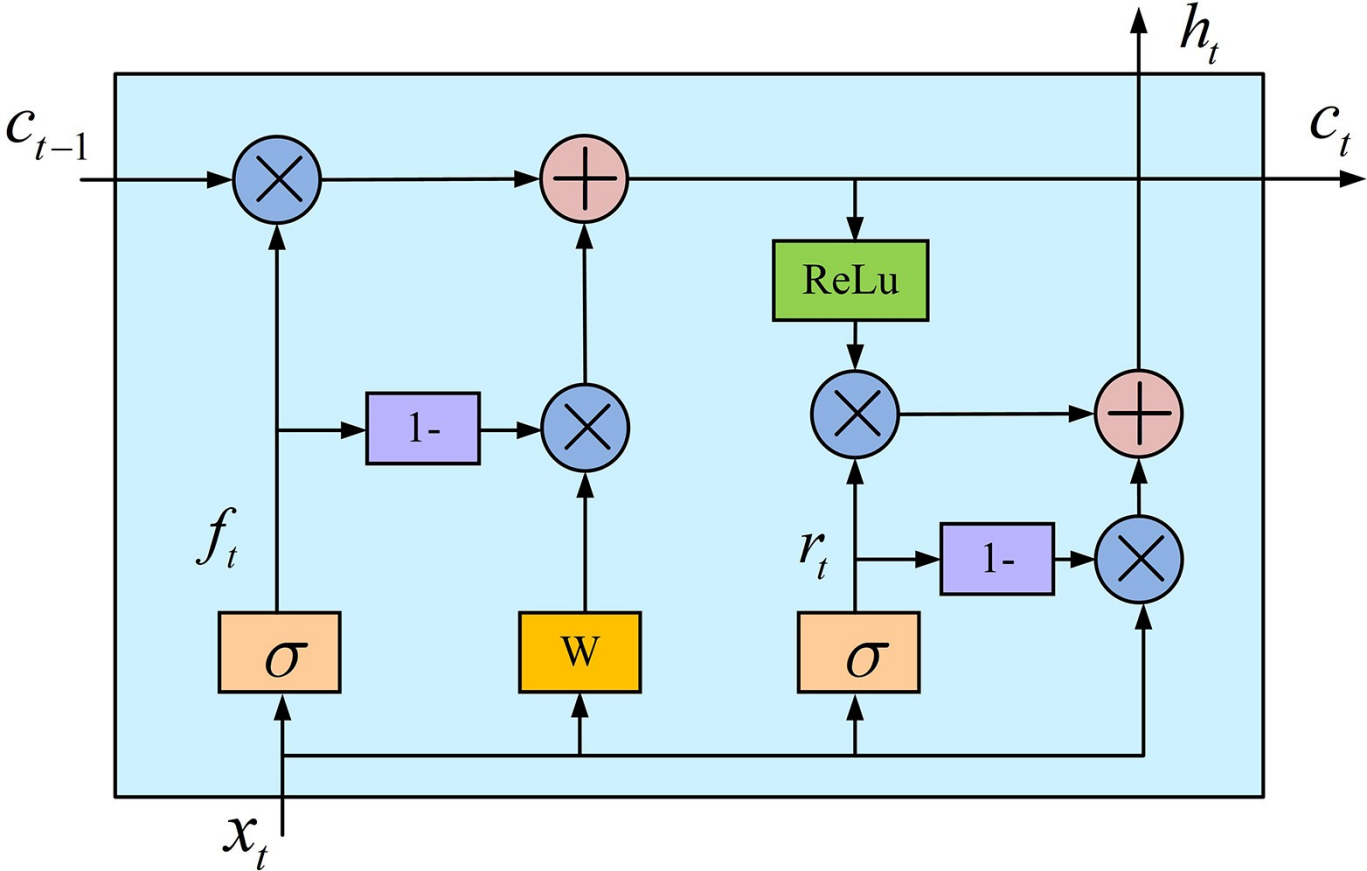
SRU neuron structure.

In Eq (9), *h*_*t*_ means the final output of the neuron at time *t*. This study builds signal modulation classification models by combining CNN with Simple Recurrent Unit (SRU) to decrease the computation of LSTM in the cycle process and simplify its network topology. The neuron structure of SRU is shown in Fig 2.

As shown in Fig 2, compared to LSTM, SRU lacks an input gate but is equipped with a reset gate. The main function of the reset gate is to skip some connections and optimize the neuron structure, thereby reducing the training time of the network. In a SRU unit, the calculation process of the forget gate is simplified, whose specific expression is shown in Eq (10) [23].


$$f_{t}=\sigma (W_{f}x_{t}+b_{f})$$ (10)

In Eq (10), *f*_*t*_, *b*_*f*_, *W*_*f*_, *x*_*t*_, and *σ* have the same meaning as in LSTM. Compared to LSTM, the forget gate in SRU is not affected by the hidden layer, and its calculation rules are simpler. The calculating formula of the reset gate is shown in Eq (11).


$$r_{t}=\sigma (W_{r}x_{t}+b_{r})$$ (11)

In Eq (11), *r*_*t*_ means the reset gate, *W*_*r*_ means the weight matrix of the reset gate, and *b*_*r*_ means the bias vector of the reset gate.

### 3.3 Construction of AMC model with CNNS and RNNS

**Fig 3:**
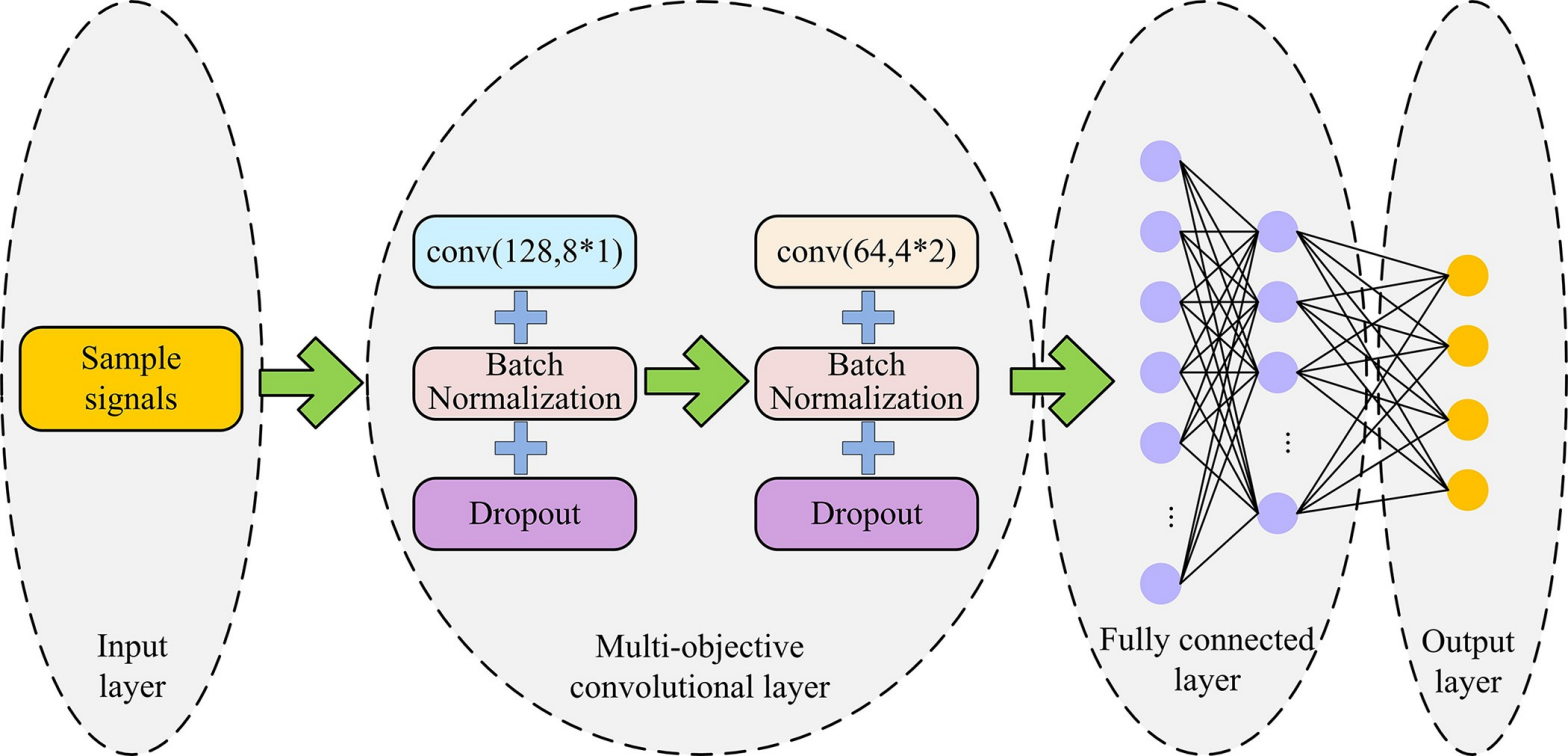
Structure of a single CNN.

The traditional AMC methods are capable of providing satisfactory signal classification outcomes when sufficient signal sample data is available. However, with the expansion in the number and types of modulation, these traditional AMC methods are unable to effectively adapt to large-scale classification tasks. To solve the above problem, this study introduces two types of neural networks in deep learning to build an optimized AMC classification model. CNN and SRU can both achieve deep extraction of signal features, and different connection ways will also cause different classification effects [24]. To build the final AMC model of signals, this study connects CNN and SRU in different ways and compares the performance of various classification models under different connection ways. First, the network structure of a single CNN is analyzed. The network structure of a single CNN is shown in Fig 3.

**Fig 4:**
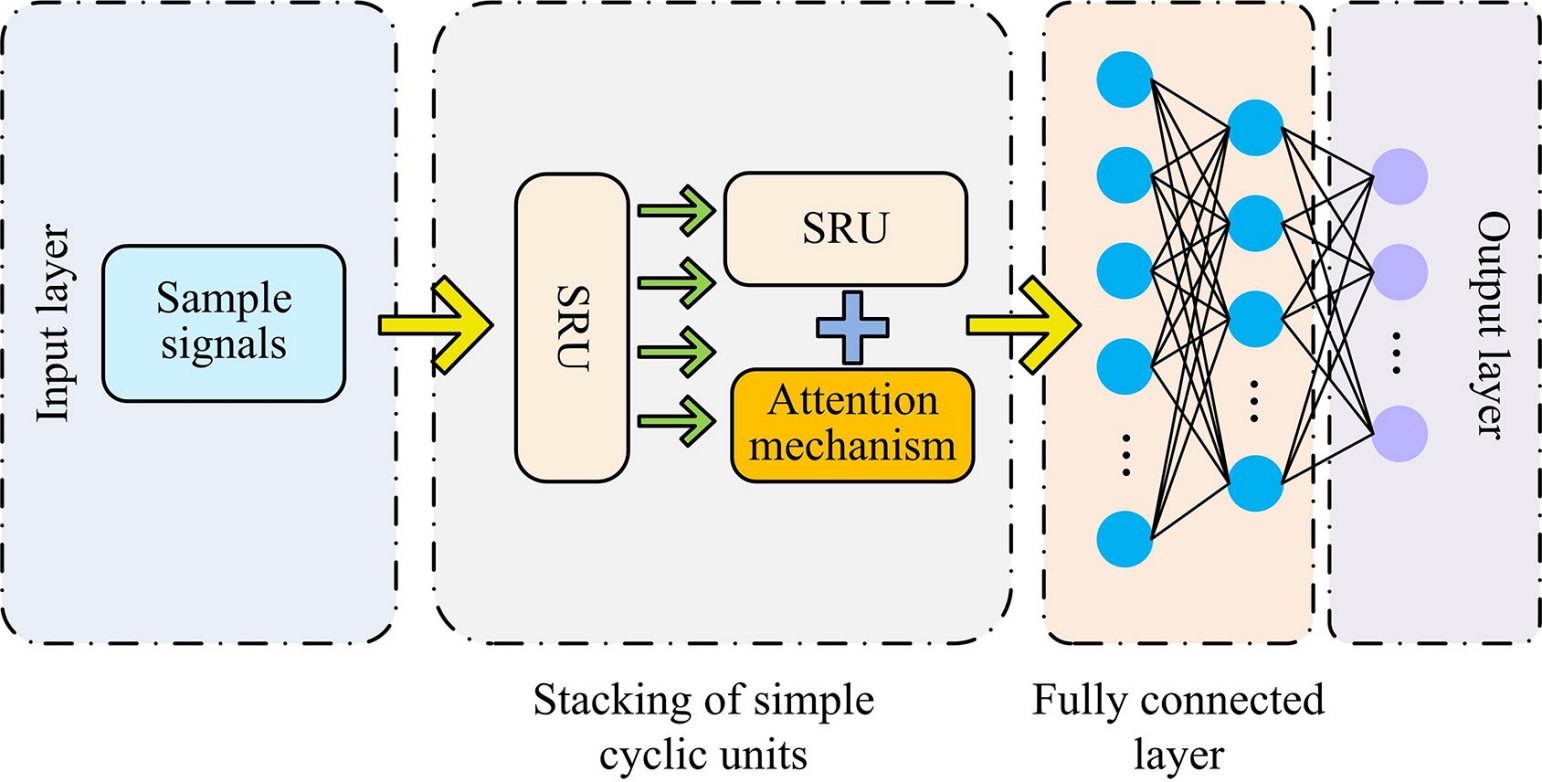
Single SRU network structure.

In Fig 3, the single CNN network structure is mainly composed of an input layer, multiple convolutional layers, a full connection layer, and an output layer. The input layer is responsible for collecting the input signal sample information, and then performing feature extraction through multiple convolutional layers. In the multiple convolutional layers, there are mainly two types of convolutional kernel for feature extraction: 8*1 and 4*2, and the number of neurons in each convolutional layer is 128 and 64 respectively. After the multiple convolutional layers complete the feature extraction, the features will be classified through the full connection layer, and the classified modulation signals will be output through the output layer. In the convolutional layer structure of a single CNN network, a Batch Normalization (BN) and a Dropout are added to accelerate the feature extraction speed of the network and prevent the model from over-fitting during training. The single SRU network structure is shown in Fig 4.

In Fig 4, the single SRU network structure mainly consists of four parts: input layer, stacked SRUs, full connection layer, and output layer. The functions of the input layer, full connection layer, and output layer in SRU are the same as those in CNN. The input layer is responsible for inputting signals. The full connection layer acts as a classifier to classify signals and output them through the output layer. The stacked SRUs in SRU are responsible for the feature extraction of signals. To increase the feature extraction accuracy of the SRU model, the study sets up a two-layer SRU structure in the stacked SRUs and optimizes it by adding an attention mechanism in the second layer of SRU.

**Fig 5:**
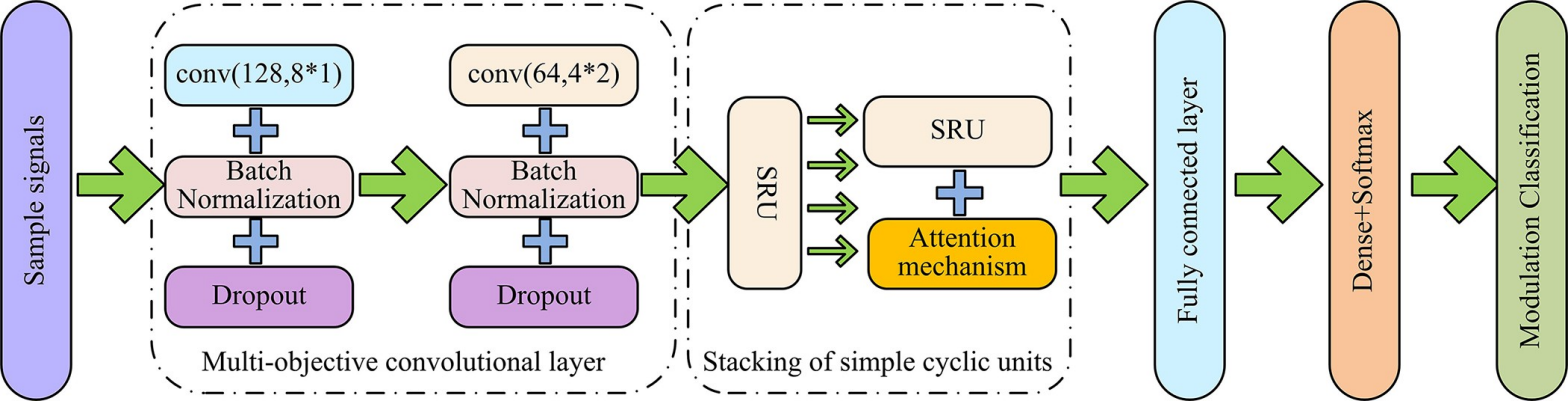
Diagram of CNN-SRU network structure.

After obtaining the single CNN and SRU network structures, different connection ways are needed to connect the two types of neural networks. The fused neural networks are used to optimize AMC, thereby enhancing its accuracy. There are three potential configurations for connecting the two neural networks. The first is to connect the SRU in series with the CNN, the second is to connect the CNN in series with the SRU, and the third is to connect the CNN and SRU in parallel [25]. The model structure diagram of CNN connected in series with SRU is shown in Fig 5.

**Fig 6:**
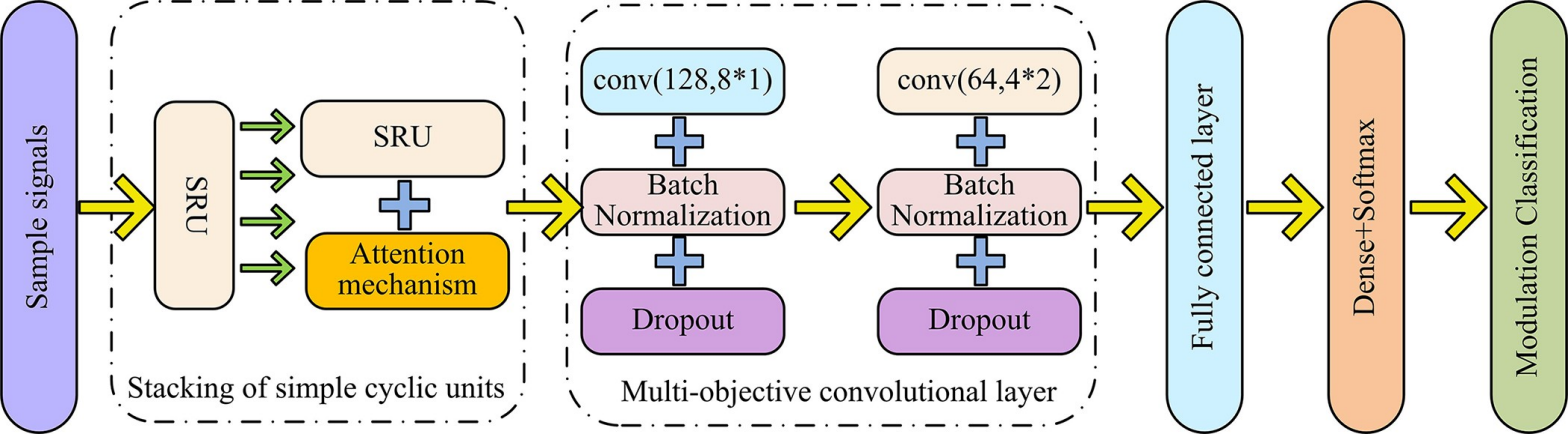
SRU-CNN network structure diagram.

In Fig 5, the signal AMC network structure under CNN connected in series with SRU is given, which is denoted as CNN-SRU in this connection mode. In the CNN-SRU network model, signal feature extraction is mainly completed by multiple convolutional layers and stacked SRUs. Signals are first extracted for features through multiple convolutional layers, and then time-series features are extracted through stacked SRUs. The model structure diagram of SRU connected in series with CNN is shown in Fig 6.

**Fig 7:**
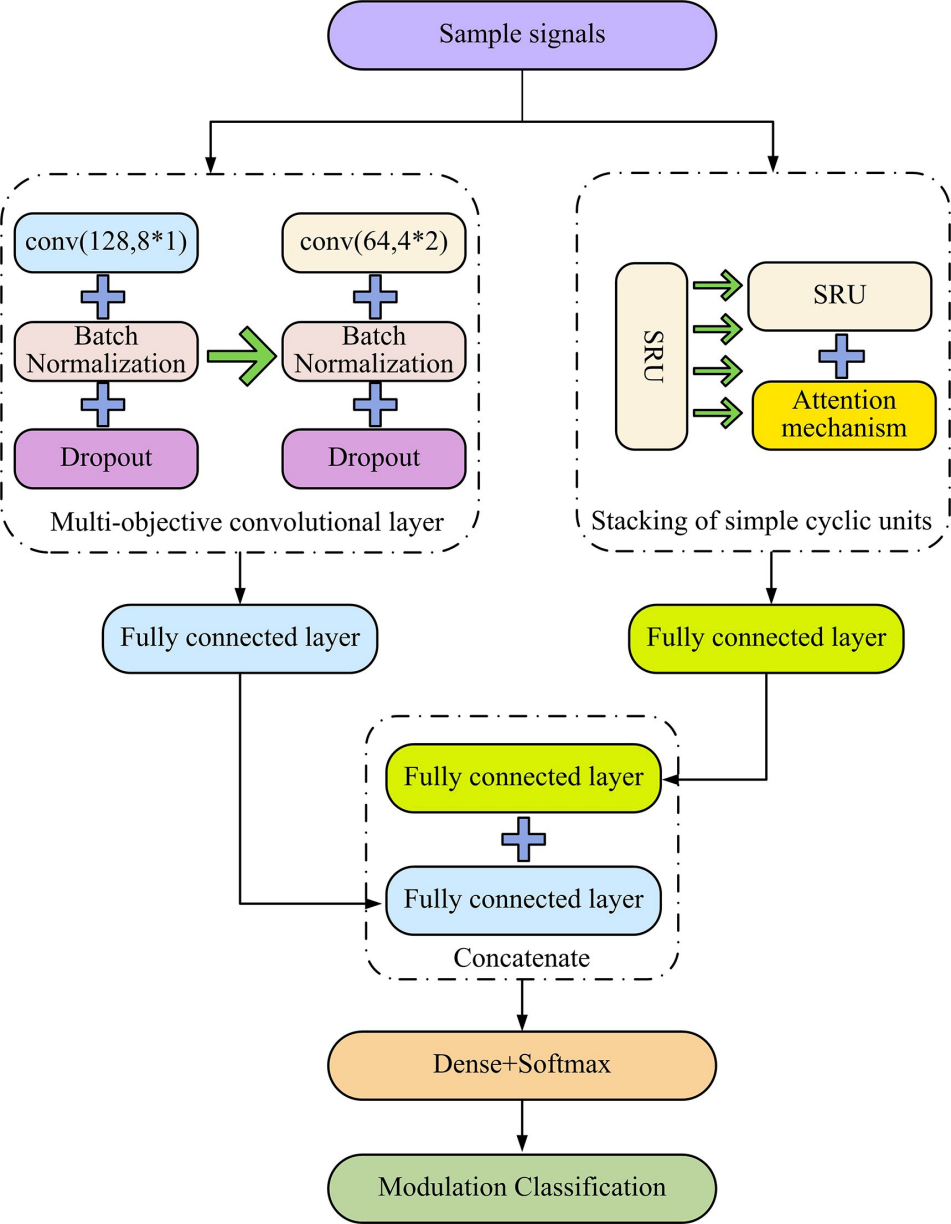
CPS network structure.

Fig 6 shows the CNN connection in series with SRU, and the network structure under this connection mode is denoted as SRU-CNN. Compared to the CNN-SRU structure, the signal in the SRU-CNN network model will first extract time-series features through stacked SRUs, and then extract remaining signal features through multiple convolutional layers. The model structure diagram obtained by connecting CNN and SRU in parallel is shown in Fig 7.

Fig 7 shows the parallel connection of CNN and SRU, which constructs a classification model. The network structure under this connection mode is denoted as CPS. In the CPS network model, the input signal is simultaneously extracted by both CNN and SRU, and then classified through a full connection layer. Additionally, an operation connection layer is added to merge the weight parameters trained by CNN and SRU, respectively. The merged weight parameters are once again passed through a full connection layer for final classification, and then the output signal is obtained.

In addition to using different connection ways to build different signal modulation classification network models, this research also needs to further construct signal models to process signal data during wireless communication, thereby facilitating feature extraction by network models. Assuming that the received baseband unknown signal during communication is *y*(*k*), its calculation expression is shown in Eq (12) [26].


y(k)=h(k)*x(k)+n(k) (12)

In Eq (12), *y*(*k*) represents the unknown baseband signal. *x*(*k*) represents the generated baseband modulated signal. *n*(*k*) represents the additive Gaussian white noise interference. *h*(*k*) represents the channel interference. Sampling the collected signal with in-phase quadrature difference causes the expression of the in-phase quadrature difference component to be Eq (13).


si=Rei+Imi (13)

In Eq (13), *s*_*i*_ represents the signal sample, Re_*i*_ represents the real part of the *i*_th feature, Im_*i*_ represents the imaginary part of the i-th feature. Under in-phase quadrature difference sampling rules, the expressions for amplitude and phase components are Eq (14) and Eq (15), respectively.


Ai=Rei2+Imi2 (14)

In Eq (14), *A*_*i*_ represents the amplitude of the i-th feature of the sample. Under in-phase quadrature difference sampling rules, the expression for feature phase is Eq (15).


θi=arctanImiRei (15)

In Eq (15), *θ*_*i*_ represents the phase value of the feature of the sample.

## 4. Performance testing and application effect analysis of signal automatic modulation model

Aiming to test the classification performance of three different connection methods, a machine learning algorithm was introduced for conducting comparative analysis. The classification accuracy, robustness, and iteration of the four algorithms under different SNRs are tested respectively. In addition, the performance of different classification models under the environment of actual channel collected data was compared, and it was ultimately found that the classification model based on the CNN-SRU connection had better classification performance.

### 4.1 Performance testing of signal AMC algorithm

To test the performance of the signal AMC algorithm, a laboratory environment was built using Python and Matlab. In Matlab, random integer functions were used to generate random data. The random data was first generated using Matlab’s random integer function, followed by the baseband signal using the modulation function. Next, the collected baseband signals were normalized and sampled by orthogonal phase difference sampling through Python. In this process, a total of 10,000 training samples were collected, which covered eight different modulation signal types and constituted the dataset for this study [27]. The combination of the power of Python and Matlab ensured that this study was able to effectively simulate a real-world signal processing environment. The flexibility of Python and the efficient numerical computational power of Matlab together enabled this study to precisely control the data generation process, thus ensuring the diversity and complexity of the dataset. This approach not only improves the quality of the datasets, but also provides a solid foundation for subsequent algorithm evaluation. The details of the generated datasets are shown in Table 1.

**Table 1:** Table of experimental data sets.

Data Set Metrics	Parameters
Modulated signal type	BPSK、QPSK、16QAM、4FSK、MSK
Modulated signal type	5
Doppler shift	100~500Hz
SNR	0~20dB
Training and validation set ratio	8:2
Training mode	Composite SNR

Table 1 gave the specific parameters of each data feature in the experimental dataset, including the type of modulation signal and the selection range of SNR. For reducing the iteration number, composite SNR training was selected in this study. The specific hardware and software configuration parameters during the experiment were shown in Table 2.

**Table 2:** Configuration.

Software and hardware	Parameters
Operating systems	Ubuntu 16.04
Processor	Intel Core i7-8700K
Memory	16GB
Graphics cards	GTX 1080 Ti
Machine learning frameworks	Tensorflow
Programming software	Python and Matlab

**Fig 8:**
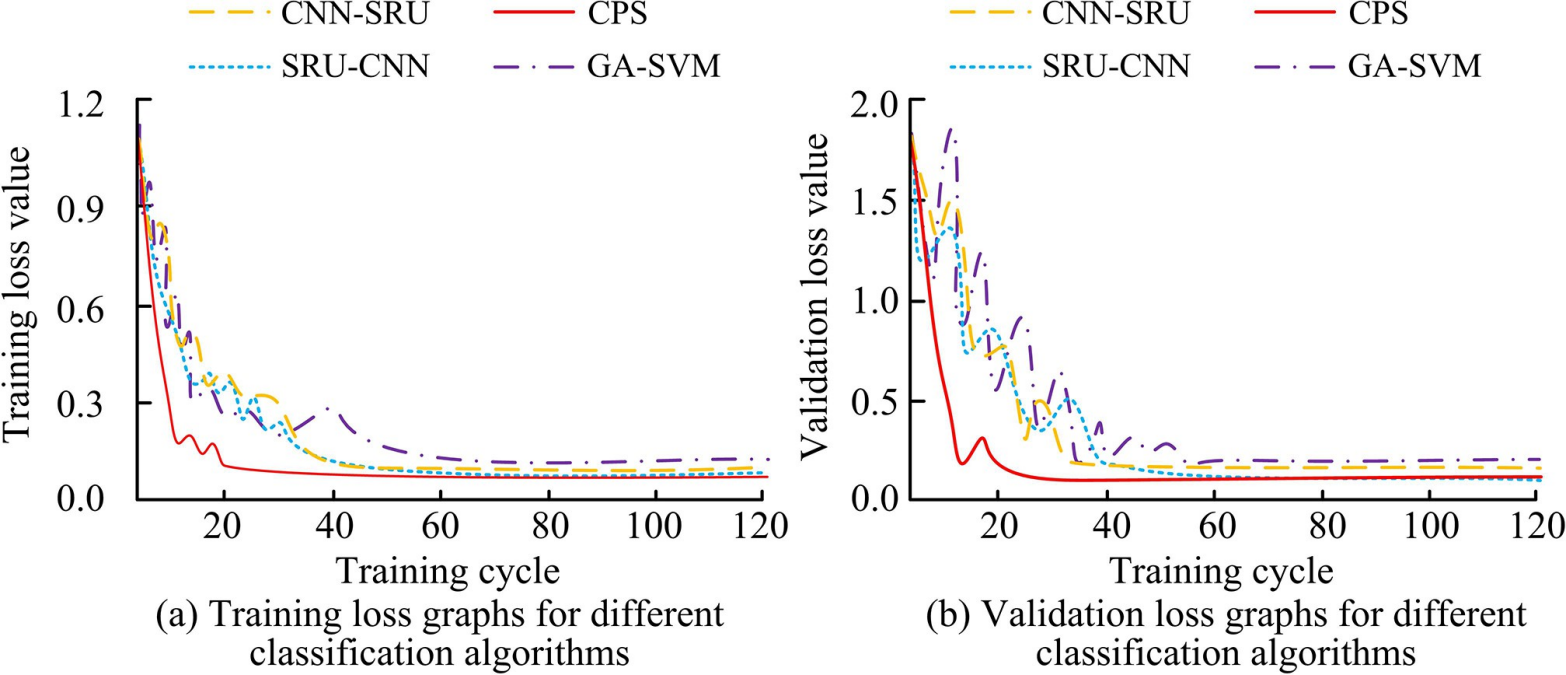
Loss profile of different fusion algorithms.

Table 2 showed the hardware and software configuration parameters for the experiment. Ubuntu 16.04 was used as the computer operating system, and the experiment was run on an Intel Core i7-8700K processor. The computer graphics card is GTX 1080 Ti and the memory was 16GB. To optimize the performance of the neural network model, the TensorFlow framework was utilized, and the key hyperparameters were adjusted. For the CNN-SRU and SRU-CNN models, the batch size was set to 64, while for the GA-SVM and CPS models, the batch size was set to 32. The learning rate for all models was initially set at 0.001 and halved after every 20 cycles. The Adam optimizer was selected for this study due to its adaptive adjustment mechanism being well-suited to the task at hand. Furthermore, the CNN-SRU and SRU-CNN models were iterated 100 times, while the GA-SVM and CPS models were iterated 150 times. The early stop strategy was implemented, whereby training was stopped if the verification loss did not improve within 10 consecutive cycles, thus preventing overfitting. These hyperparameter settings were designed to balance training efficiency with maximum classification accuracy to ensure robust performance of the model at all SNRs. The genetic algorithm optimization of support vector machine (GA-SVM) was introduced as a comparison. To test loss curves of CNN-SRU, SRU-CNN, CPS, and GA-SVM under different training periods, the experiment was conducted, whose results were shown in Fig 8.

Fig 8(A) and 8(B) illustrate the loss curves of the four fusion algorithms on the training and validation datasets, respectively. As shown in Fig 8(A), when the training period was 20, CPS exhibited the fastest convergence to a stable state, with a training loss value of 0.13. At the training periods of 42, 50, and 64, the CNN-SRU, SRU-CNN, and GA-SVM algorithms reached a stable state, with stable training loss values of 0.14, 0.13, and 0.15, respectively. Fig 8(B) showed that when the training period was 29, CPS iterated to a stable state, with a validation loss value of 0.18. When the training periods were 33, 45, and 58, CNN-SRU, SRU-CNN, and GA-SVM iterated to stable states, with stable validation loss values of 0.25, 0.17, and 0.28, respectively. The training time of different algorithms could be well compared using the loss curve graph. When the iteration number is shorter, the algorithm can be trained to the steady state more quickly. In addition, the shape of the loss curve, the fluctuations and the number of cycles it needs for iterating to the steady state reflect the convergence speed and stability of the algorithm. For example, the loss curve of the CPS algorithm quickly reached a lower loss value in fewer training cycles due to its network structure and optimization strategy that allowed the model to converge faster. Compared to CNN-SRU, SRU-CNN, and GA-SVM, the CPS algorithm outperformed the other algorithms in terms of stability and convergence speed. This difference was due to the fact that CPS was constructed with a concurrent structure that was more suitable for handling the given dataset and hence its training strategy was more effective.

**Fig 9:**
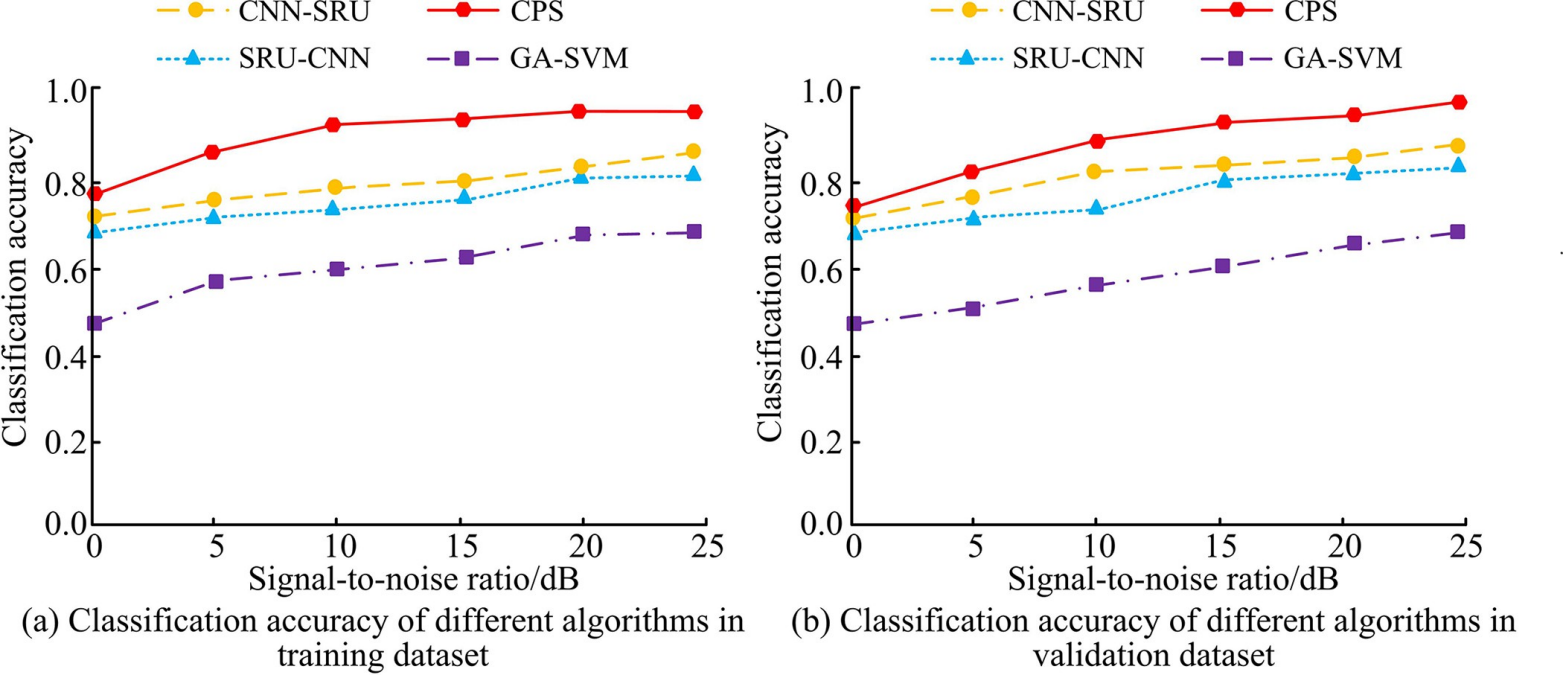
Classification accuracy of different fusion algorithms.

Fig 9(A) and 9(B) show the classification accuracy of the four fusion algorithms on the training and validation datasets, respectively. As shown in Fig 9(A), with the increase of SNRs, the classification accuracy of CPS, CNN-SRU, SRU-CNN, and GA-SVM fusion algorithms all increased. When the SNR was 25dB, the classification accuracy values of CPS, CNN-SRU, SRU-CNN, and GA-SVM on the training dataset were the highest, which were 0.98, 0.89, 0.82, and 0.65, respectively. As shown in Fig 9(B), when the SNR was 25dB, the classification accuracy values of CPS, CNN-SRU, SRU-CNN, and GA-SVM on the validation dataset were also the highest, which were 0.99, 0.88, 0.84, and 0.64, respectively. According to the given results, the classification accuracy of all four algorithms showed an increasing trend as the SNR increased. This indicated that the SNR is a key factor affecting the classification performance. At higher SNRs (e.g., 25 dB), the algorithms were able to distinguish different modulation types more effectively because the signal was of higher quality with less noise interference. The CPS algorithm showed the highest classification accuracy in both the training and validation datasets (up to 0.98 and 0.99, respectively), which may be attributed to the CPS algorithm’s superior learning and generalization capabilities when dealing with specific datasets. In contrast, the GA-SVM exhibited the lowest classification accuracy (0.65 and 0.64, respectively), which was likely due to the fact that support vector machines were not as flexible as neural network-based methods in dealing with complex nonlinear patterns. Additionally, the CPS algorithm connects CNNs and SRUs in parallel, which may allow the algorithm to capture the spatio-temporal features of the signals more efficiently, thus improving the classification accuracy. The series approach of CNN-SRUs and SRU-CNNs may be limited in terms of capturing specific types of features, resulting in a slightly lower performance than the CPS. To summarize, different SNRs have an impact on the classification performance of the algorithms. Overall, when the SNR is larger, the algorithm’s classification performance is better.

**Fig 10:**
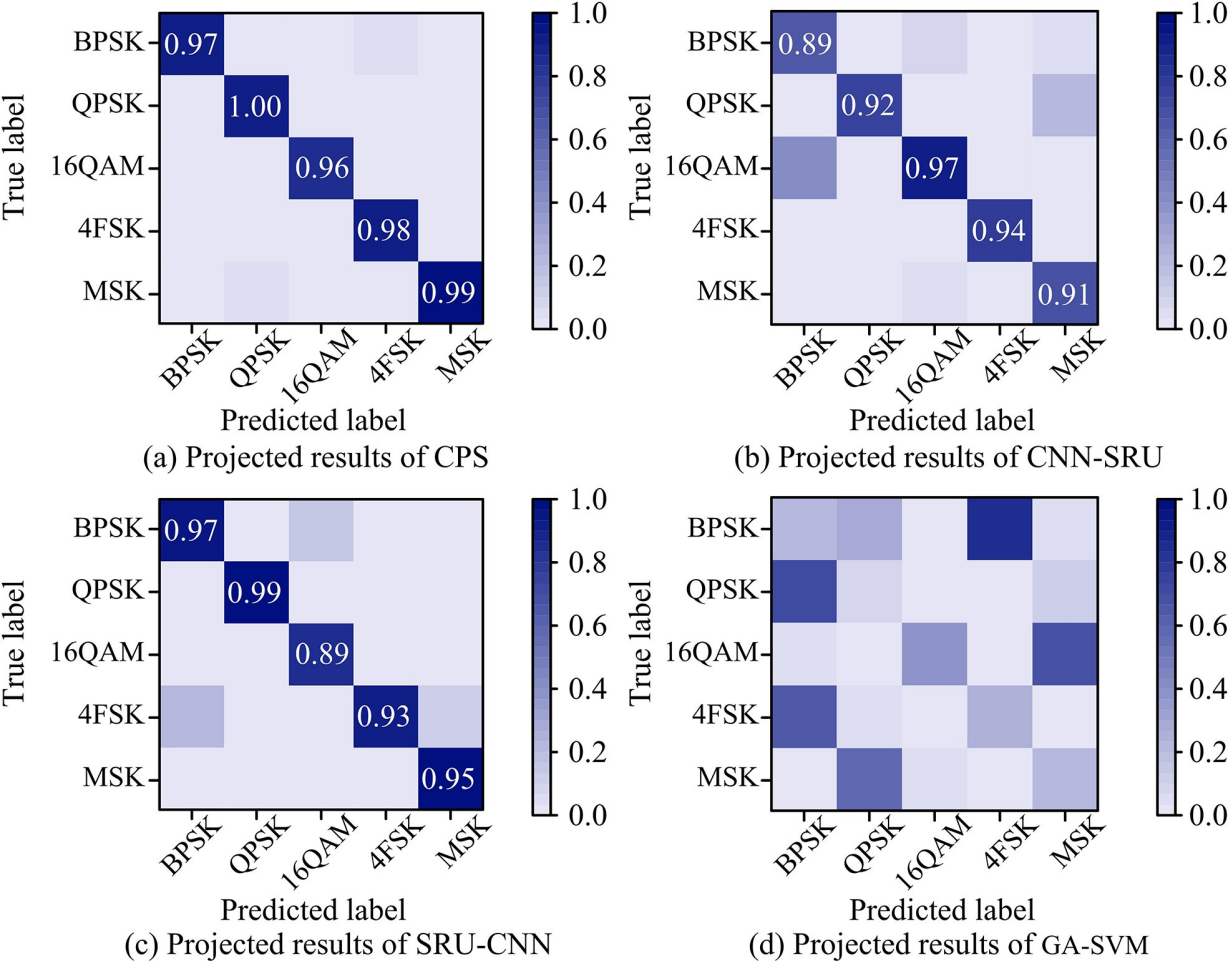
Confusion matrix for different fusion algorithms at 25dB.

Fig 10 presented the confusion matrices of the four fusion algorithms under 25dBSNRs. BPSK, QPSK, 16QAM, 4FSK, and MSK were selected as labels for five types of modulation signals, and the predicted results of the for tested algorithms were obtained. In Fig 10(A), CPS achieved an accuracy rate of over 0.95 for accurately predicting all five types of modulation signals, and there was no other missed classification behavior. In Fig 10(B), CNN-SRU achieved an accuracy rate of over 0.85 for predicting all five types of modulation signals, but there was missed classification behavior for BPSK and MSK signals. In Fig 10(C), SRU-CNN has an accuracy rate of over 0.85 for predicting all five types of modulation signals, but there was missed classification behavior for BPSK, 16QAM, and MSK signals. As shown in Fig 10(D), GA-SVM could not effectively predict the five types of modulation signals, and there was a lot of misclassification behavior in this classification method.

**Fig 11:**
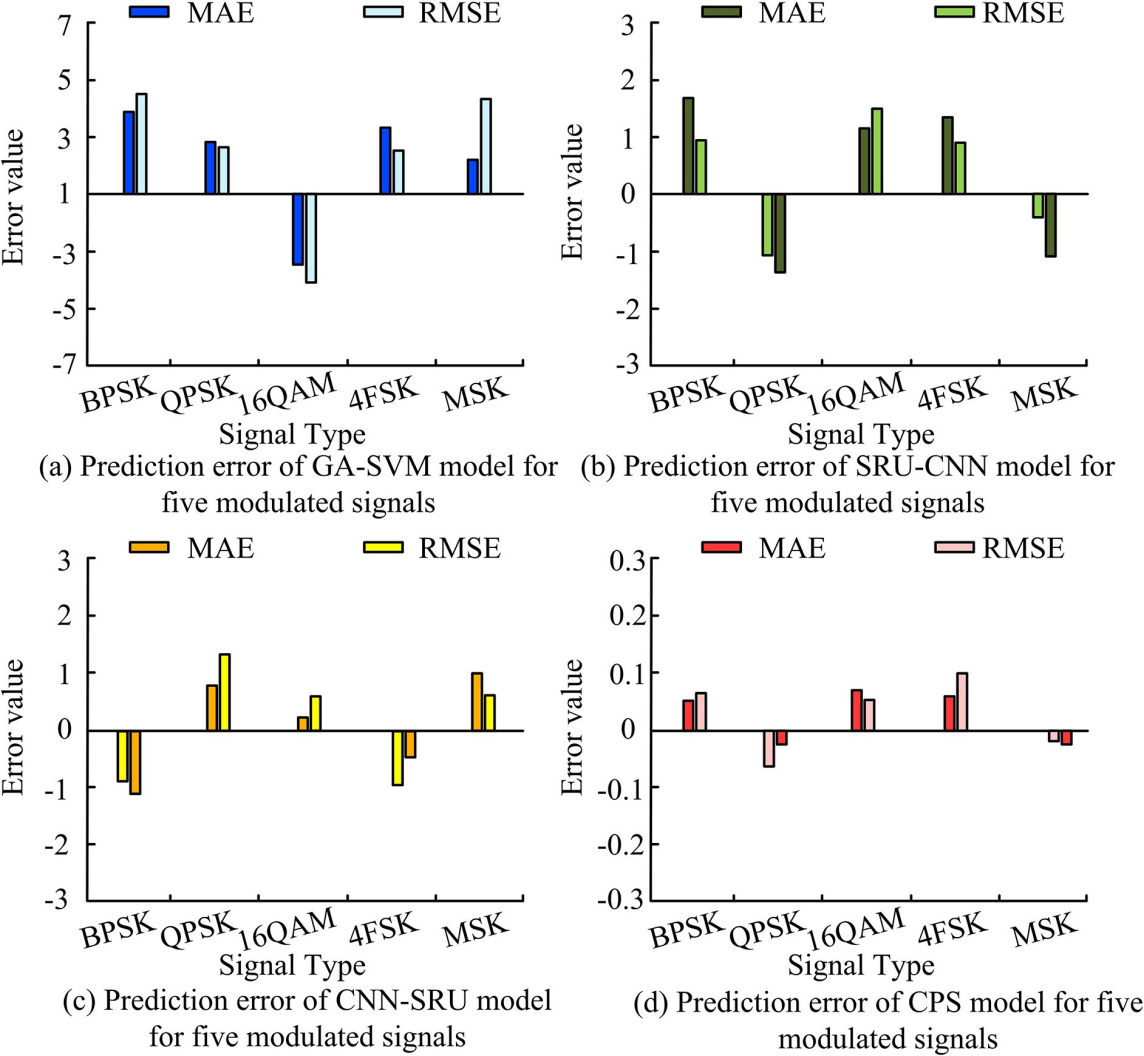
Prediction errors of four models for five modulated signals.

Fig 11 presented the prediction errors of the four algorithms for five types of modulation signals. The Mean Absolute Error (MAE) and root mean square error (RMSE) were selected as error metrics. As shown in Fig 11(A), the MAE and RMSE values of GA-SVM for the five types of modulation signals remained between -5 and 5. As shown in Fig 11(B) and 11(C), the MAE and RMSE values of SRU-CNN and CNN-SRU for the five types of modulation signals remained between -2 and 2, but the error fluctuations of CNN-SRU were smaller than those of SRU-CNN. As shown in Fig 11(D), the MAE and RMSE values of CPS for the five types of modulation signals remained between -0.1 and 0.1. In summary, CPS achieved better error performance and could more accurately detect different modulation signals.

### 4.2 Application effect of signal AMC model

**Fig 12:**
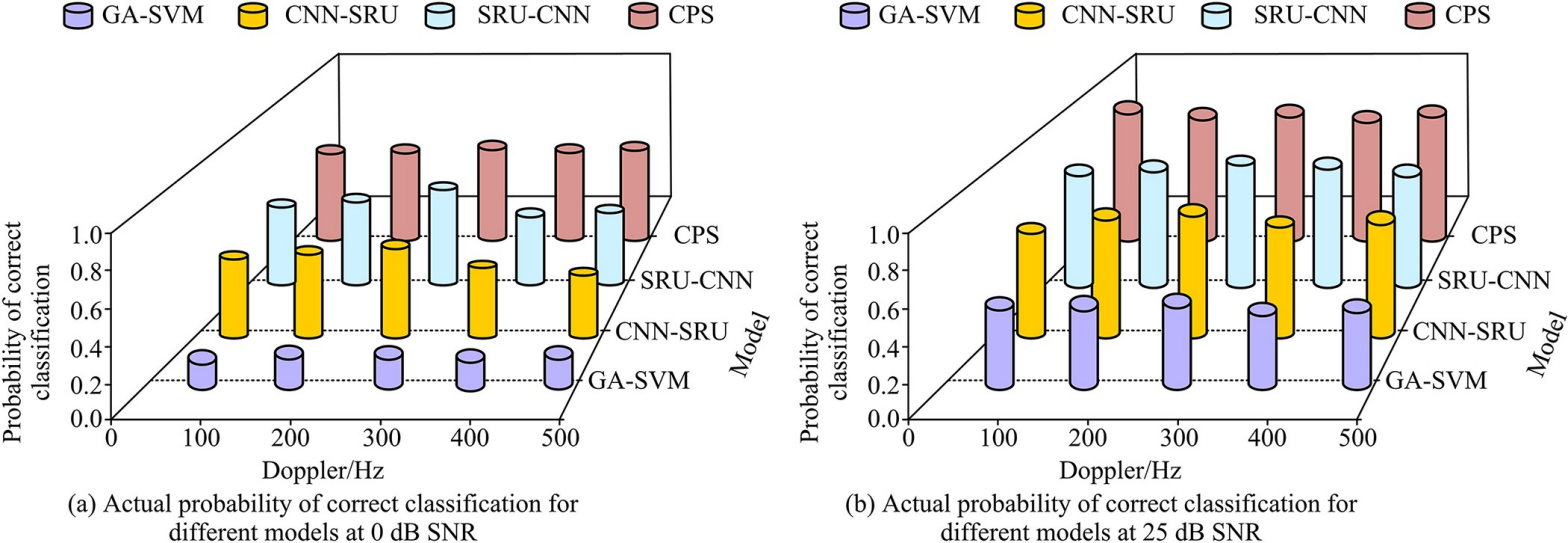
Probability of correct classification for classification models with different SNRs.

After testing the performance of the four fusion algorithms, CPS was found with the best performance in modulation signal classification. In practical applications, Doppler frequency shift could affect the performance of the network and thus affect the signal classification effect. Therefore, it was necessary to test the actual classification effect of the four classification models under different Doppler frequency shift conditions. In the experiment, the four models was varied with Doppler frequency shift conditions under different SNRs. The correct classification probabilities under this condition were shown in Fig 12.

Fig 12 showed the correct classification probability of the four models varying with Doppler frequency shift conditions under different SNRs. As shown in Fig 12(A), when the SNR was 0dB, the correct classification probability of the GA-SVM classification model was the lowest. When the Doppler frequency shift value increased from 0Hz to 500Hz, the correct classification probability of the GA-SVM classification model remained stable at around 0.3, while the correct classification probability of the CPS classification model remained stable at around 0.6. Conversely, the correct classification probabilities of CNN-SRU and SRU-CNN first increased, then decreased, and finally they remained stable. Among them, the highest correct classification probabilities of CPS, CNN-SRU, and SRU-CNN under 0dB were 0.66, 0.61, and 0.63, respectively. In Fig 12(B), when the SNR was 25dB, the correct classification probabilities of all four models were higher than those under 0dB. The highest correct classification probabilities of CPS, CNN-SRU, SRU-CNN, and GA-SVM under 25dB were 0.98, 0.90, 0.89, and 0.62, respectively. In the high SNR (e.g., 25 dB) condition, the probability of correct classification for all models was higher than that in the low SNR (e.g., 0 dB) condition. This indicated that the SNR was an important factor in determining the classification accuracy of the models. Under the condition of high signal quality, the models were able to recognize the modulated signals more accurately. In addition, the Doppler shift affected different models to different degrees. For example, the classification probability of CNN-SRU and SRU-CNN increased and then decreased when the Doppler shift value increased, and then stabilized. In contrast, the classification probability of the CPS model remained relatively stable under changes in Doppler shift values. This may have reflected the greater robustness of the CPS model in dealing with frequency variations. Under different SNR and Doppler shift conditions, the CPS model showed a high classification probability. Especially under high SNR conditions, its correct classification probability was close to 0.98. This indicated that the CPS model had a superior performance in processing high-quality signals. In contrast, the GA-SVM model exhibited a lower classification probability under all conditions, particularly under low SNR, with a classification probability of approximately 0.3. This may indicate that the GA-SVM model has a limited ability to process complex or low-quality signals. An increase in Doppler frequency shift value will have a certain impact on the classification model, particularly for CNN-SRU and SRU-CNN.

Table 3 displays the classifying accuracy of the four models under different Doppler frequency shift conditions and SNRs. As demonstrated in Table 3, an increase in SNR from 0 dB to 25 dB has been observed to result in an enhancement in the classification accuracy of all four models. The results demonstrated that, with an increase in Doppler frequency shift conditions from 100 Hz to 500 Hz, the CNN-SRU, SRU-CNN, and GA-SVM models achieved the highest classification accuracy at 300 Hz, while the CPS model exhibited no change in classification accuracy regardless of the Doppler frequency shift conditions. In summary, compared to CNN-SRU and SRU-CNN, which were serial structures, CPS achieved higher classification accuracy with its parallel approach to build a signal modulation classification model. Compared to the machine classification optimization model GA-SVM, the CNN-SRU, SRU-CNN, and CPS models, which used neural network fusion algorithms to build, had better classification effects.

**Table 3:** Classification accuracy of the four models under different Doppler shift conditions.

Model	SNR	Doppler shift of the frequency		
		100Hz	300Hz	500Hz
CPS	0dB	0.64	0.65	0.66
	15dB	0.81	0.82	0.83
	25dB	0.97	0.98	0.98
CNN-SRU	0dB	0.53	0.61	0.59
	15dB	0.75	0.79	0.73
	25dB	0.87	0.90	0.84
SRU-CNN	0dB	0.55	0.63	0.58
	15dB	0.79	0.82	0.77
	25dB	0.83	0.89	0.85
GA-SVM	0dB	0.32	0.35	0.34
	15dB	0.48	0.55	0.43
	25dB	0.59	0.62	0.55

In Table 4, the classification times of the four models, CPS, CNN-SRU, SRU-CNN, and GA-SVM, were given for different combinations of center frequency/bandwidth. The main objective of this experiment was to evaluate the effectiveness of AMC-based neural network fusion algorithms for AMC, especially in low SNR and multipath propagation environments. The smaller bandwidths (i.e., 20 MHz and 40 MHz) were chosen for the experiment based on several considerations. The first objective was to control the complexity of the experiment. The smaller bandwidth helped to reduce the complexity of the experimental setup, allowing for a more focused evaluation of the algorithmic performance, with less emphasis on external environmental disturbances. The second objective was to improve the adaptability of the algorithm. By testing under narrower bandwidth conditions, the adaptability and robustness of the algorithms could be better evaluated when facing spectrum limitations in practical applications. Finally, the objective was to save computational resources. Data processing at smaller bandwidths required less computational resources, which was important for applications on real-time or low-power devices. In summary, although wider bandwidths can theoretically provide more information, in the context of this research, the smaller bandwidths were chosen to ensure that the experiments were manageable and the algorithms were practical. In this way, it enabled this study to effectively evaluate and validate the performance of the proposed AMC neural network fusion algorithm under different signal conditions, while ensuring that the implementation of the experiments was practical.

**Table 4:** Classification times of the four models at different central frequencies and bandwidths.

Center frequency / bandwidth	CPS	CNN-SRU	SRU-CNN	GA-SVM
2 GHz / 20 MHz	0.35s	0.46s	0.52s	0.60s
2 GHz / 40 MHz	0.41s	0.55s	0.55	0.67s
4 GHz / 20 MHz	0.31s	0.44s	0.56s	0.65s
4 GHz / 40 MHz	0.38s	0.59s	0.58s	0.70s
6 GHz / 20 MHz	0.41s	0.51s	0.62s	0.75s
6 GHz / 40 MHz	0.49s	0.66s	0.69s	0.79s

In Table 4, when the center frequency/bandwidth was 4 GHz / 20 MHz, CPS and CNN-SRU had the shortest classification time, which was 0.31s and 0.44s, respectively. When the center frequency/bandwidth was 2 GHz / 20 MHz, SRU-CNN and GA-SVM had the shortest classification time, which was 0.52s and 0.60s, respectively. In general, with the increasing values of center frequency/bandwidth, the classification performance of all four models increased, but CPS and CNN-SRU showed the maximum value at a center frequency/bandwidth value of 4 GHz/20 MHz. CPS had the shortest classification time for all center frequency/bandwidth types. This outcome proved that CPS performed a faster classification speed compared to the other three classification methods.

The classification accuracy of the four models in classifying different waveform is shown in Table 5. In Table 5, the classification accuracy of the CPS model in classifying the four modulated waveform of PAM, QAM, BPSK, and QPSK was 0.94, 0.95, 0.97, and 0.99, respectively. The classification accuracy of the CNN-SRU model in classifying the four modulated waveform of PAM, QAM, BPSK, and QPSK was 0.88, 0.89, 0.93, and 0.92, respectively. The classification accuracy of SRU-CNN model in classifying the four modulated waveform of PAM, QAM, BPSK, and QPSK was 0.85, 0.88, 0.91, and 0.89, respectively. The classification accuracy of GA-SVM model in classifying the four modulated waveform of PAM, QAM, BPSK, and QPSK was 0.81, 0.84, 0.85, and 0.86, respectively. This showed that the CPS model also performed better than the other three models in classifying different modulated waveform.

**Table 5:** Classification accuracy of the four models when classifying different waveforms.

Modulation type	CPS	CNN-SRU	SRU-CNN	GA-SVM
PAM	0.94	0.88	0.85	0.81
QAM	0.95	0.89	0.88	0.84
BPSK	0.97	0.93	0.91	0.85
QPSK	0.99	0.92	0.89	0.86

Table 6 illustrates the classification accuracy of the four models under varying SNRs, ranging from -10dB to 0dB. As shown in Table 6, an increase in SNR from -10dB to 0dB resulted in enhanced signal classification accuracy for all four models. Among these models, CPS exhibited the highest accuracy, with the greatest improvement in accuracy observed. At a SNR of -10dB, the classification accuracy of CPS, CNN-SRU, SRU-CNN, and GA-SVM was 0.68, 0.55, 0.58, and 0.61, respectively. At an SNR of 0 dB, the classification accuracy of CPS, CNN-SRU, and GA-SVM was 0.68, 0.55, 0.58, and 0.61, respectively. The classification accuracy of CPS, CNN-SRU, SRU-CNN, and GA-SVM was 0.95, 0.81, 0.85, and 0.86, respectively.

**Table 6:** Classification accuracy of four models under low SNR.

SNR	CNN-SRU	SRU-CNN	GA-SVM	CPS
-10	0.55	0.58	0.61	0.68
-9	0.58	0.59	0.63	0.71
-8	0.61	0.61	0.66	0.73
-7	0.64	0.63	0.68	0.78
-6	0.67	0.65	0.72	0.79
-5	0.71	0.67	0.75	0.83
-4	0.72	0.68	0.76	0.87
-3	0.75	0.72	0.78	0.89
-2	0.78	0.76	0.81	0.91
-1	0.79	0.83	0.84	0.93
0	0.81	0.85	0.86	0.95

**Fig 13:**
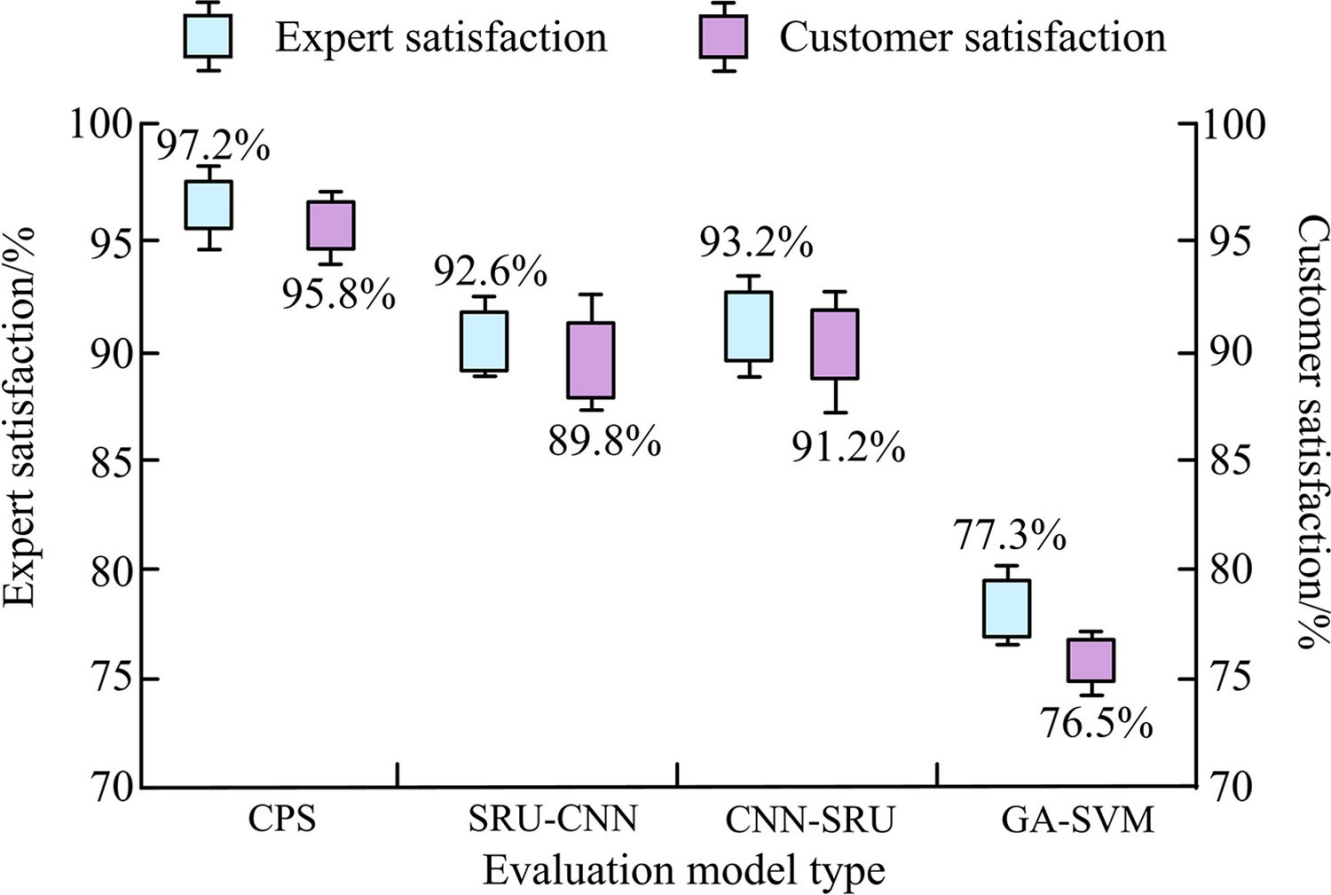
Satisfaction of experts and users with classification models.

Fig 13 presents the satisfaction of experts and users for the four classification models. Both experts and users agreed that the CPS classification model, which used a parallel structure, had better classification effects. Therefore, this model received a satisfaction rate of 97.2% from experts and 95.8% from users. In contrast, GA-SVM only received a satisfaction rate of 77.3% from experts and 76.5% from users.

## 5. Discussion

The findings of this study were compared with those of previously published relevant literature, demonstrating the advantages and innovations of current methods in AMC. Kim et al. enhanced feature recognition by converting signal features into images and employing image processing techniques. In contrast, this study reduced the potential for information loss during feature conversion by enhancing the representation of signals directly in the frequency domain. When the SNR was 25 dB and the Doppler shift was 500 Hz, the classification accuracy of the model in this study was as high as 0.98, while the accuracy of the method proposed in literature [28] was lower than this value. This indicated that the proposed model exhibits superior performance at higher SNR, which is primarily attributed to the strategy of direct feature enhancement and optimization in the frequency domain, which mitigates the loss incurred during the process of information conversion. Furthermore, Fu et al. [29] effectively reduced the communication overhead by implementing a combination of distributed learning and ensemble learning. However, this study directly improved the accuracy and efficiency of classification by improving the algorithm structure itself, thereby reducing complexity and computational requirements without sacrificing performance. Finally, in comparison to the study conducted by Zhou et al. [30] within the context of small-sample learning, the proposed method not only demonstrated satisfactory performance under low-sample conditions, but also exhibited enhanced robustness under low SNR through the implementation of novel learning strategies. While the model designed by Zhou et al. exhibits superior performance under certain conditions, the approach proposed in this study maintained a high level of performance over a wider range of SNRs. In conclusion, this study has made a significant contribution to the field of deep learning for AMC. In particular, it has demonstrated the potential and practicality of the proposed approach in complex environments, such as those characterised by SNR changes and limited sample size.

## 6. Conclusion

To optimize traditional AMC technology, CNN and SRU were connected using three different methods. Ultimately three signal AMC models were built, namely CPS, CNN-SRU, and SRU-CNN. The performance test of the built models showed that CPS achieved stable status after training 20 times and validating 29 times on the training and validation datasets, with significantly fewer training iterations than CNN-SRU, SRU-CNN, and GA-SVM. In addition, the classification accuracy of the four fusion algorithms increased with the increase of SNRs. When the SNR was 25dB, the highest classification accuracy of CPS, CNN-SRU, SRU-CNN, and GA-SVM on the training dataset was 0.98, 0.89, 0.82, and 0.65, respectively, while on the validation dataset, it was 0.99, 0.88, 0.84, and 0.64, respectively. Furthermore, comparing the confusion matrices of the four hybrid algorithms, it was found that CPS had an accuracy rate of over 0.95 for identifying five types of modulation signals (BPSK, QPSK, 16QAM, 4FSK, and MSK). Nevertheless, GA-SVM was unable to effectively identify the five modulation signal types. Finally, the Doppler frequency shift condition was introduced to test the practical application of the four models in signal classification. The correct classification probability of CNN-SRU and SRU-CNN first increased then decreased and finally remained stable as the Doppler frequency shift value increased. The correct classification probability of GA-SVM and CPS was unaffected by the Doppler frequency shift value and remained stable throughout. Finally, the CPS classification model received a satisfaction rate of 97.2% from experts and 95.8% from users. In summary, the designed neural network hybrid classification model can improve the classification effect of AMC on modulation signals. In terms of the future research, as many derived models of RNN exists, the proposed model is considered to be combined with other RNN models for further improvement.

## Supporting information

S1 Dataset (DOC)
