# Supplementary material for: Signal automatic modulation based on AMC neural network fusion
Source: PLoS One. 2024 Jun 6;19(6):e0304531. doi: 10.1371/journal.pone.0304531 (PMC11156377; doi:10.1371/journal.pone.0304531)
Supplement: S1 Dataset — (DOC) [file pone.0304531.s001.doc]

**Figure 1 Structure of LSTM neuron**

No data

**Figure 2 SRU neuron structure**

No data

**Figure 3 Structure of a single CNN**

No data

**Figure 4 Single SRU network structure**

No data

**Figure 5 Diagram of CNN-SRU network structure**

No data

**Figure 6 SRU-CNN network structure diagram**

No data

**Figure 7 CPS network structure**

No data

**Figure 8 Loss profile of different fusion algorithms**

| Loss value | Number of samples | CNN-SRU | SRU-CNN | GA-SVM | CPS |
| --- | --- | --- | --- | --- | --- |
| Training loss | 20 | 0.38 | 0.33 | 0.23 | 0.13 |
|  | 40 | 0.16 | 0.19 | 0.29 | 0.13 |
|  | 60 | 0.14 | 0.13 | 0.18 | 0.13 |
|  | 80 | 0.14 | 0.13 | 0.15 | 0.13 |
|  | 100 | 0.14 | 0.13 | 0.15 | 0.13 |
|  | 120 | 0.14 | 0.13 | 0.15 | 0.13 |
| Validation loss | 20 | 0.78 | 0.81 | 0.57 | 0.26 |
|  | 40 | 0.25 | 0.21 | 0.26 | 0.18 |
|  | 60 | 0.25 | 0.17 | 0.28 | 0.18 |
|  | 80 | 0.25 | 0.17 | 0.28 | 0.18 |
|  | 100 | 0.25 | 0.17 | 0.28 | 0.18 |
|  | 120 | 0.25 | 0.17 | 0.28 | 0.18 |

**Figure 9 Classification accuracy of different fusion algorithms**

| Data set | Number of samples | CNN-SRU | SRU-CNN | GA-SVM | CPS |
| --- | --- | --- | --- | --- | --- |
| Training dataset | 0 | 0.76 | 0.67 | 0.49 | 0.78 |
|  | 5 | 0.78 | 0.71 | 0.57 | 0.87 |
|  | 10 | 0.79 | 0.73 | 0.58 | 0.92 |
|  | 15 | 0.81 | 0.75 | 0.61 | 0.93 |
|  | 20 | 0.83 | 0.79 | 0.63 | 0.97 |
|  | 25 | 0.89 | 0.82 | 0.65 | 0.98 |
| Validation dataset | 0 | 0.75 | 0.68 | 0.47 | 0.76 |
|  | 5 | 0.78 | 0.70 | 0.51 | 0.84 |
|  | 10 | 0.82 | 0.71 | 0.56 | 0.89 |
|  | 15 | 0.83 | 0.78 | 0.60 | 0.92 |
|  | 20 | 0.85 | 0.81 | 0.62 | 0.96 |
|  | 25 | 0.88 | 0.84 | 0.64 | 0.99 |

**Figure 10** Confusion matrix for different fusion algorithms at 25dB

| Model | Modulation signals | BPSK | QPSK | 16QAM | 4FSK | MSK |
| --- | --- | --- | --- | --- | --- | --- |
| CPS | BPSK | 0.97 | 0.02 | 0.07 | 0.13 | 0.03 |
|  | QPSK | 0.03 | 1.00 | 0.06 | 0.02 | 0.01 |
|  | 16QAM | 0.08 | 0.05 | 0.96 | 0.01 | 0.00 |
|  | 4FSK | 0.05 | 0.06 | 0.05 | 0.98 | 0.01 |
|  | MSK | 0.06 | 0.15 | 0.11 | 0.04 | 0.99 |
| CNN-SRU | BPSK | 0.89 | 0.11 | 0.23 | 0.10 | 0.12 |
|  | QPSK | 0.23 | 0.92 | 0.16 | 0.08 | 0.41 |
|  | 16QAM | 0.48 | 0.28 | 0.97 | 0.17 | 0.08 |
|  | 4FSK | 0.19 | 0.09 | 0.05 | 0.94 | 0.05 |
|  | MSK | 0.25 | 0.15 | 0.19 | 0.06 | 0.91 |
| SRU-CNN | BPSK | 0.97 | 0.13 | 0.26 | 0.09 | 0.14 |
|  | QPSK | 0.16 | 0.99 | 0.15 | 0.08 | 0.17 |
|  | 16QAM | 0.21 | 0.11 | 0.89 | 0.10 | 0.16 |
|  | 4FSK | 0.45 | 0.07 | 0.09 | 0.93 | 0.38 |
|  | MSK | 0.18 | 0.11 | 0.12 | 0.09 | 0.95 |
| GA-SVM | BPSK | 0.36 | 0.42 | 0.26 | 0.86 | 0.22 |
|  | QPSK | 0.65 | 0.30 | 0.23 | 0.18 | 0.31 |
|  | 16QAM | 0.29 | 0.09 | 0.42 | 0.19 | 0.75 |
|  | 4FSK | 0.58 | 0.15 | 0.09 | 0.38 | 0.23 |
|  | MSK | 0.21 | 0.47 | 0.17 | 0.22 | 0.34 |

**Figure** 11 Prediction errors of four models for five modulated signals

| Model | Modulation signals | MAE | RMSE |
| --- | --- | --- | --- |
| CPS | BPSK | 0.5 | 0.6 |
|  | QPSK | -0.2 | -0.6 |
|  | 16QAM | 0.7 | 0.5 |
|  | 4FSK | 0.6 | 0.9 |
|  | MSK | -0.1 | -0.2 |
| CNN-SRU | BPSK | -1.2 | -0.8 |
|  | QPSK | 0.6 | 1.4 |
|  | 16QAM | 0.3 | 0.5 |
|  | 4FSK | -0.5 | -1.1 |
|  | MSK | 0.8 | 0.6 |
| SRU-CNN | BPSK | 1.7 | 0.8 |
|  | QPSK | -1.4 | -1.1 |
|  | 16QAM | 1.2 | 1.5 |
|  | 4FSK | 1.3 | 0.7 |
|  | MSK | -1.2 | 0.4 |
| GA-SVM | BPSK | 3.8 | 4.5 |
|  | QPSK | 2.6 | 2.3 |
|  | 16QAM | -3.5 | -4.2 |
|  | 4FSK | 3.7 | 2.2 |
|  | MSK | 1.8 | 4.3 |

**Figure 12 Probability of correct classification for classification models with different SNRs**

| Signal-to-noise ratio | Doppler/Hz | CNN-SRU | SRU-CNN | GA-SVM | CPS |
| --- | --- | --- | --- | --- | --- |
| 0dB | 100 | 0.53 | 0.55 | 0.32 | 0.64 |
|  | 200 | 0.61 | 0.63 | 0.35 | 0.63 |
|  | 300 | 0.59 | 0.58 | 0.34 | 0.65 |
|  | 400 | 0.52 | 0.55 | 0.36 | 0.62 |
|  | 500 | 0.50 | 0.51 | 0.32 | 0.66 |
| 25dB | 100 | 0.87 | 0.83 | 0.59 | 0.97 |
|  | 200 | 0.88 | 0.88 | 0.61 | 0.96 |
|  | 300 | 0.90 | 0.89 | 0.62 | 0.98 |
|  | 400 | 0.87 | 0.86 | 0.60 | 0.97 |
|  | 500 | 0.84 | 0.85 | 0.55 | 0.98 |

**Figure 13 Satisfaction of experts and users with classification models**

| Satisfaction | CNN-SRU | SRU-CNN | GA-SVM | CPS |
| --- | --- | --- | --- | --- |
| Expert satisfaction | 93.2% | 92.6% | 77.3% | 97.2% |
| Customer satisfaction | 91.2% | 89.8% | 76.5% | 95.8% |
